# Not worth watching? Examining gender bias, perceptions of ability, and consumer behaviour for the Women’s Australian Football League

**DOI:** 10.1080/00049530.2025.2543796

**Published:** 2025-08-13

**Authors:** Mackenzie R. Glazbrook, Sarven S. McLinton, Stephanie N. Webb, Mikaela S. Owen

**Affiliations:** Justice and Society, University of South Australia, Adelaide, Australia

**Keywords:** Australian sport, essentialism, gender bias, sports consumers, Women’s sport

## Abstract

**Objective:**

Despite the increasing presence of women in professional sport, gender biases continue to shape consumer perceptions and engagement. This study examines how gendered attitudes influence evaluations of athlete quality and, in turn, consumer intentions towards the AFLW.

**Method:**

A community sample (N = 862) completed an online survey assessing gender attitudes, and beliefs about sport and masculinity, and perceptions of athlete quality in football.

**Results:**

Multigroup path analysis revealed differences in consumer perceptions of women’s and men’s football. Traditional attitudes towards gender and masculinity were significantly related to negative perceptions of women footballers’ abilities, which were also associated with reduced desire to watch and attend AFLW matches.

**Conclusions:**

These results highlight the role of gender biases in shaping public engagement with women’s sport. Addressing these biases, particularly in media representation and marketing, may be key to increasing consumer investment in women’s football and promoting greater gender equity in sport.

## KEY POINTS

**What is already known about this topic**:

1. Despite the growth in popularity of women’s sport, negative consumer attitudes towards women athletes remain prevalent.
2. Conservative gender role beliefs contribute to negative attitudes towards women athletes, limiting support for women’s professional sport
3. Perceived athlete quality and entertainment are key factors shaping consumer interest in sport.

**What this topic adds**:

1. This study provides insight into how gender-biased attitudes shape perceptions of women’s athletic abilities and impact consumer engagement.
2. Findings highlight the interplay between gender norms, sport and masculinity attitudes, and consumer attitudes, demonstrating that conservative attitudes towards gender and sport were related to lower engagement with women’s football.
3. The study provides practical recommendations for sport governing bodies and marketers, emphasising the role of visibility, media representation, and inclusive engagement strategies in challenging entrenched biases and increasing support for the AFLW.

Stereotypes and bias about the physical capabilities of women and girls are entrenched in the world of sport (Allison, 2020). Despite the increasing participation of women in traditionally male-dominated sports, particularly professional leagues, dominant constructions of gender continue to reify men’s superiority in this space (Allison, 2020). These beliefs are rooted in gender essentialism, which presumes innate, biologically determined differences in ability between men and women (Greene, 2021), casting women as naturally less capable athletes. Such ideology tends to facilitate discriminatory practice in professional sporting leagues, often translating to inadequate funding, limited media attention, and inequitable resource distribution for women’s sport (Mohapatra, 2021; Olgilvie & McCormack, 2021) – including in Australian Rules football (hereafter, “football”). These inequalities not only limit opportunities for women’s sport development but may also shape broader public perceptions of their athletic capabilities, reinforcing barriers to engagement and support.

Launched in 2017 amongst a broader rise in women’s semi-elite and elite women’s sports leagues in Australia, the Women’s Australian Football League (AFLW) became the premier national competition for women’s football (Burke et al., 2023). The league launched with just eight teams, expanding to 18 by 2022 to match the men’s league (AFLM), following substantial grassroots interest and growth in women’s football participation (AFL, 2022). Despite this expansion, the AFLW continues to face structural and financial constraints, including shorter seasons, part-time contracts, and reduced access to training and support resources compared to the AFLM (Klugman et al., 2022; Vinall, 2023). These inequities reflect broader trends across women’s professional sport in Australia, which, while rapidly expanding, remains under-resourced relative to men’s leagues in terms of funding, media visibility, and institutional support (Lebel et al., 2021).

A key rationale for such structural discrepancies is the perception that women’s football lacks consumer interest and therefore commercial value. This logic, however, evokes a cyclical pattern in which limited investment may contribute to enduring perceptions of women’s sport as inferior. Although the AFLW’s introduction challenged the culture of masculinity in Australian sport, the years since the league’s inauguration have highlighted persistent consumer biases in women’s football, with the AFLW attracting a limited fanbase compared to the AFLM (Vinall, 2023). Whilst factors such as inadequate marketing, media attention and investment are important considerations, engagement differences are often attributed to the alleged poorer quality of women’s football (Glazbrook et al., 2024). These perceptions can be partly contextualised by the relative newness of the AFLW and the ongoing development of professional talent pathways. Restricted long-term access to elite training environments has shaped the depth of the current talent pool, contributing to unfair comparisons between the AFLW and the more established, fully professional AFLM. Moreover, the AFLW’s relatively rapid expansion has been questioned; in contrast, other women’s leagues (e.g., the National Rugby League Women’s) have advocated for a slower expansion to ensure playing quality could develop in step with talent and infrastructure growth (Taylor et al., 2022).

However, emerging research in sport contexts where women’s leagues have received longer-term institutional support (e.g., soccer) suggests that negative evaluations of women’s professional sport cannot be attributed solely to league maturity or developmental stage. Rather, they are also shaped by biased attitudes towards women athletes, particularly essentialist beliefs about their supposed physical inferiority (Gomez-Gonzalez et al., 2024; Lebel & Danylchuk, 2009). These attitudes may influence how consumers evaluate the quality and entertainment capacity of women’s sport (Allison, 2018), arguably reducing the likelihood of audience engagement. Given consumer interest and investment are key components of the growth, sustainability, and viability of sport organisations (Giachino et al., 2024), the aim of the current study is to build on existing research into attitudes towards women’s sport by examining the relationship between gender bias, perceptions of athlete quality, and consumer behaviour in women’s football.

## Gender essentialism

Unfavourable stereotypes about women athletes are particularly prevalent in traditionally masculine sports such as football, which is characterised by intensity, pressure, and heavy contact, without the use of protective equipment (Nicholson et al., 2021). This emphasis on physicality and aggression fuels perceptions of football as an unsuitable sport for women for reasons relating to (1) infantilising concern for their physical safety, and (2) stereotypes surrounding the athletic capacities of the female body (Goorevich & LaVoi, 2024; Olgilvie & McCormack, 2021; Ross & Shinew, 2008). These beliefs extend from essentialist ideology which purports the existence of innate, rigid gender/sex categories and traits, where athleticism is associated predominantly with masculinity (Allison & Love, 2023). Essentialism refers to a broad range of perspectives and theories that describe differences between categories or groups as fixed, inherent characteristics (Greene, 2021; Messner, 2011). In the context of gender, essentialism extends biological determinism, asserting that behavioural and social role differences between women and men can be explained solely through binary biological mechanisms (Greene, 2021).

Essentialist ideology is institutionalised in sport through structural mechanisms such as gender segregation, which reinforce the idea that women require separate, often lesser, spaces to compete, thereby justifying the ongoing treatment of women’s sport as secondary (Anderson, 2008). Moreover, when women athletes do perform to a high standard, essentialist assumptions often invite alternative, delegitimising explanations for success. For example, research indicates that elite women may face accusations of doping or challenges to their sex, including transphobic claims that seek to invalidate their success (Bernache-Assollant et al., 2024; Messner, 1988; Mohapatra, 2021). Given the most popular entertainment sports tend to be characterised by physicality, and risk (e.g., football), facilitating equal opportunity in participation and career for women in these spaces continues to be challenging. Women are more readily accepted in sports aligned with aestheticism or grace, which tend to offer fewer professional pathways and receive significantly less media attention and investment (Allison & Love, 2023). In this way, essentialist assumptions continue to shape not only participation and representation, but also public perceptions of legitimacy, entertainment value, and the broader worth of women’s sport.

Feminist scholars have long critiqued gender essentialism as a reductive framework which obscures the complex socio-cultural and intersectional dimensions of gender (LaVoi & Goorevich, 2024; Allison, 2018). Such critiques are supported by multidisciplinary research which demonstrates that gender differences in performance exist along a continuum shaped more by opportunity, training, and environment than by sex at birth (Cooky, 2009; Kane, 1995). Essentialist claims about inherent gender difference are not only empirically unsubstantiated, but also disproportionately reflect the experiences of white, cisgender, heterosexual, economically privileged individuals – thus obscuring intersectional realities and reinforcing exclusionary norms under the guise of objectivity (LaVoi & Goorevich, 2024). While essentialist frameworks may be used in attempts to promote gender equity (e.g., by emphasising “feminine” strengths), they risk entrenching the binary logics and hierarchies they seek to dismantle (LaVoi & Goorevich, 2024). As Goorevich et al. (2025) note, essentialist beliefs endure not because they are valid, but because they serve to justify and sustain inequalities and gender hierarchy in sport. In this paper, we do not endorse essentialist views of gender, but rather, adopt an essentialist framework to understand how such beliefs shape perceptions of women’s inferiority in sport.

### Sociocultural influences and gender essentialist beliefs in sport

Olgilvie and McCormack (2021) described the concept of “gender-essentialist narratives” which emphasise how stereotypes are facilitated by a number of sociocultural influences, which may overtly and/or subtly communicate that sport is inherently masculine. In an Australian context, organised sport has a pervasive influence in engraining gendered beliefs, shaping social norms regarding not only sport participation, but also the categorisation of specific sports as either feminine (e.g., netball) or masculine (e.g., football; Booth et al., 2023). From an early age, boys are encouraged to view sports participation as integral to masculinity, while it is generally considered socially acceptable for girls to withdraw from organised sport (Drummond et al., 2022). With fewer girls encouraged to participate, the resulting underrepresentation of women in these spaces reinforces stereotypes about their supposed lack of ability. For example, microaggressions such as “you run/throw/kick like a girl” are pervasive in grassroots sports, embedding beliefs about women’s supposed lack of physical prowess from a young age (Kaskan & Ho, 2016). These biases are amplified through media coverage which consistently portrays women’s sport as less entertaining and valuable than men’s sport (Musto et al., 2017). Through both negative framing and exclusion, audiences are primed to perceive men’s sport as inherently more exciting (Lebel & Danylchuk, 2009; Olgilvie & McCormack, 2021).

Exposure to these ideologies – whether explicit or implicit – has enduring consequences for individual aspirations and broader societal perceptions (Cerbara et al., 2022). For example, Persson (2023) found that engrained gender ideologies in youth sports influence how both children and parents perceive the physical abilities of girls and boys. The findings suggested that girls viewed mens’ soccer as more exciting and important than women’s soccer, leading to the perception that grassroots boys’ soccer deserves greater investment. Moreover, even high-performing girls seemed to evaluate their abilities by comparing themselves to boys (e.g., being “as good as a boy”), rather than challenging notions of girls’ athletic inferiority (Persson, 2023). These findings illustrate how exposure to stereotypes portraying women and girls as inherently weaker not only shape sports participation experience but also perpetuate essentialist beliefs that position men’s sports as superior. Consequently, these attitudes likely also influence how women’s sports are consumed and valued by the public.

## Gender bias in perceptions of women’s sport

Relevant research has consistently identified a number of important motivational factors sports consumer engagement, yet such findings have scarcely been applied to the context of women’s professional sport. In broader sports consumer research (i.e., men’s sport), external motivators such as advertising, media coverage, and promotional efforts have been found to play a pivotal role in driving fan engagement (Paek et al., 2021). Moreover, entertainment, drama, athlete quality, and celebrity status of players/athletes act as important motivators of engagement with sport (Funk et al., 2022; Koronios et al., 2020; Mayer & Hungenberg, 2020; Rizvandi et al., 2019). In football specifically (i.e., AFLM), engagement may also be shaped by long-standing team loyalty, tradition (e.g., family influence), social connection, and a perceived sense of belonging (Duncan, 2016).

Whilst these factors are important to understanding sports fandom more broadly, women’s sport faces unique challenges in cultivating similar forms of engagement. Although the factors influencing fan engagement with women’s sport remain underexplored, existing findings demonstrate that women’s leagues in traditionally male-dominated sports are often considered less dramatic, less exciting, and more boring than men’s sports (Fink, 2015; Lebel & Danylchuk, 2009; Williams et al., 2022). Given the importance of these elements for attracting sports fans, such perceptions are likely to inhibit consumer interest. However, there is evidence to suggest that consumer perceptions of women’s sport may be shaped more by gendered stereotypes rather than the actual quality of play. For example, Gomez-Gonzalez et al. (2024) found that when obscuring their gender, the performance of women and men soccer players was rated equally, yet when gender was visible, men received higher evaluations than women. Similarly, Angelini (2008) found no physiological differences participants’ arousal levels when watching women’s versus men’s sport, despite participants self-reporting lower levels of excitement whilst watching women’s – suggesting that perceived differences in entertainment value may be socially constructed rather than reflective of actual experience.

Extending this insight, Fink et al. (2016) demonstrated that even when men acknowledged the competence of women in participatory sport contexts, they continued to rely on stereotypes that positioned women’s sport as less athletic or entertaining. Even attempts to compliment women athletes often invoked gendered comparisons (e.g., “they don’t play like girls”), reinforcing essentialist beliefs about women’s inferiority. As Messner (1988, p. 205) described, this form of discourse operates as a “double-edged sword”, where praise couched in comparison ultimately sustains the very stereotypes it attempts to challenge. As such, even when women athletes defy expectations, their achievements are frequently downplayed or invalidated (Olgilvie & McCormack, 2021).

## The current study

Although the increasing presence of women in competitive sports has ostensibly challenged traditional perceptions of athleticism (Mohapatra, 2021; Olgilvie & McCormack, 2021), the intersection of industry inequalities and entrenched gender stereotypes continues to shape the way women’s sports are perceived. Such biases appear to lead consumers to undervalue the quality and spectacle of women’s sports, contributing to reduced fan engagement and support. Therefore, the fundamental aim of this research is to explore gender bias in perceptions of athlete quality in the context of women’s football, and how this relates to consumer behaviour:

**RQ**: What is the relationship between perceptions of gender attitudes, perceived athlete quality, and consumer intentions?

**Figure 1.**
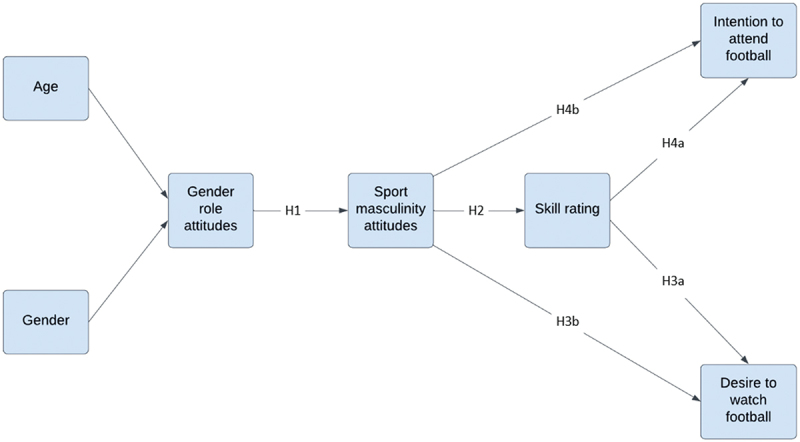
This figure depicts the conceptual path model and hypothesised regression paths.

To address this, we first explore group differences in how participants rate the quality of AFL players. For the main portion of analysis, we posit that the relationship follows a specific sequence; that demographic characteristics inform our gender attitudes, and those general attitudes inform more domain-specific sport-based attitudes, which then impacts on our evaluation of player skills, and in turn, our intended actions/motivation to watch that sport. As such, multigroup path analysis was conducted to explore how gendered attitudes influence perceptions of the quality of women’s football, and in turn, how these perceptions may be associated with consumer behaviour. The following hypotheses are proposed (also visually depicted in Figure 1):

H1: There will be a positive relationship between gender role attitudes and sport masculinity attitudes, such that those with more traditional gender role attitudes will also hold more conservative attitudes towards sport and masculinity.

H2: Sport masculinity attitudes will be negatively associated with player skill ratings, with those holding more conservative attitudes towards sport and masculinity providing lower evaluative scores for women players compared to those with more egalitarian attitudes.

H3: Higher evaluative scores for the women players will be associated with an increased desire to watch AFLW (H3a), and conservative attitudes towards sport and masculinity will be associated with a decreased desire to watch AFLW (H3b).

H4: Higher evaluative scores for the women players will be associated with higher intention to attend AFLW matches (H4a), and conservative attitudes towards sport and masculinity will be associated with a lower intention to attend AFLW matches (H4b).

## Method

### Sample and data collection

A total of 1439 participants began the online survey, which was administered via the web-based software, Qualtrics between July 2023 and January 2024. Before commencing the survey, participants confirmed their eligibility (i.e., Australian residents aged 18+) and provided consent to participate. Ethical approval was granted by the relevant Human Research Ethics Committee (protocol #204864) prior to collection of data. Recruitment predominantly occurred via social media platforms (i.e., Facebook, Instagram), through both paid advertising (i.e., Meta ads; Your customers are here. Find them with Meta ads, 2024) and snowballing methodology. A total of 272 participants were excluded from the sample due to incomplete responses (i.e., did not complete the survey beyond key data collection points), and an additional 305 participants were removed following bot detection protocol. Non-binary participants were also excluded from final analyses due to inadequate sample size. The final sample comprised N *=* 862 participants (see Table 1 for sample demographics).

**Table 1.** Demographic overview of the final sample.

Variable	Mean	SD	*N*	% of *N*
Age (range 18–85)	55.97	14.86	862	
Gender				
Women			296	34.3
Men			558	64.7
Gender diverse^a^			8	0.9
Sexual orientation				
Heterosexual			709	82.2
Sexual minorities^b^			153	17.7
Location				
Australian Capital Territory			16	1.9
New South Wales			108	12.5
Northern Territory			8	0.9
Queensland			159	18.4
South Australia			215	24.9
Tasmania			36	4.2
Victoria			255	29.5
Western Australia			65	7.5

*Note*. ^a^ Gender diverse includes transgender and non-binary people.

^a^Sexual minorities include bisexual, homosexual, and pansexual.

### Design

This study employed a between-subjects experimental design, implemented through an online survey. The survey was constructed to obtain a detailed overview of attitudes towards both the women’s and men’s Australian Football Leagues, incorporating measures of participant demographic information, football fandom, and gender role attitudes.

**Figure 2.**
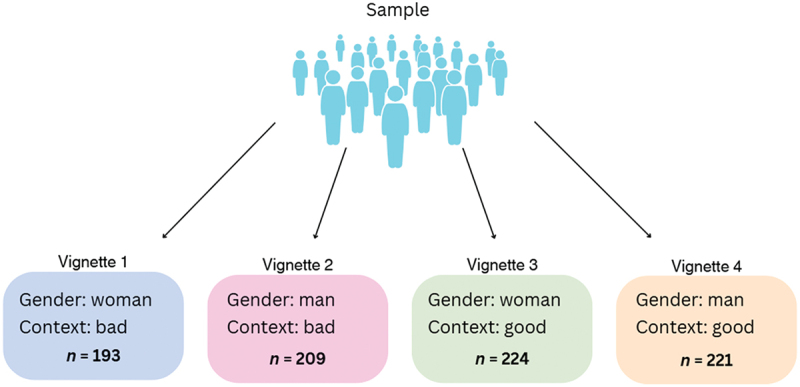
Vignette contexts and sub-sample sizes.

In the experimental portion of the survey, participants were assigned to one of four vignettes using the random allocation function in Qualtrics. The vignettes were developed by the research team based on common terminology and phrasing found within match reports and commentary from the AFL. Each vignette described the performance of a fictional player, and the environmental conditions of the match played, with variations based on player gender (i.e., woman or man), and match performance (i.e., good vs bad) to assess participant attitudes in various contexts (see Figure 2 for an overview and sub-sample sizes). While the vignettes included football specific details and statistics, they were written to be accessible to individuals with minimal knowledge of football, allowing them to deduce the quality of the player (see Appendix for sample vignette). To mitigate priming effects, player gender was not explicitly stated, and gender-neutral names (i.e., Alex/Sam) were used. However, binary pronouns were included throughout the vignettes (i.e., she/her, he/him) to subtly indicate the gender of the player. Following the presentation of the vignette, participants rated the quality of the player’s football skills on a scale from: 1 (very poor) to 6 (excellent).

#### Measures

The survey measures detailed below were presented in the same order to all participants, following demographic items, with responses measured on a 6-point forced choice scale unless otherwise specified (i.e., 1 = strongly disagree − 6 = strongly agree).

##### Football Fandom

Using a single item indicator, participants were asked about their status as fans of football as a sport (i.e., “Please rate the degree to which you consider yourself a fan of Australian Rules Football”; rated from 1 = not at all to 6 = dedicated fan). To ensure consistency amongst responses, participants were provided with conceptual definitions of both non-fans and dedicated fans. The vignettes were presented following this item, and prior to the remaining scales to avoid priming.

##### Heteronormative & Gender Normative Sport Attitudes

Gender normativity and heteronormativity in sport attitudes were assessed using the 8-item sexism, sport masculinity, and homophobia subscale (extracted from the Multidimensional Sport Attitudes Scale; Yakut et al., 2016). Designed for adaptability, the scale was modified in this study to assess attitudes towards participation in masculinely perceived contact sports (e.g., “women should not play men’s sports such as football or rugby”). Items were also modified to measure sports consumer perspectives, rather than the perspective of athletes (e.g., “it disturbs me when gay, lesbian, or bisexual athletes represent my team in a sport competition”). Reliability analyses demonstrated good internal consistency (α = .86) in this sample.

##### Desire to Watch Football

Football fandom was further contextualised through assessment of desire to watch football, using a modified version of the 7-item Desire to Watch Sports subscale (from the Multidimensional Sport Attitudes Scale; Yakut et al., 2016), for women’s and men’s football respectively (e.g., “I would watch [women’s/men’s] AFL even if my favourite team was not playing”). Reliability analyses demonstrated excellent internal consistency (AFLW: α = .92; AFLM: α = .90) in this sample.

##### Gender Role Attitudes

Gender role attitudes were assessed using the 13-item Social Roles Questionnaire (SRQ; Baber & Tucker, 2006). This scale measures the degree to which gender is associated with specific societal roles (8- item gender linked subscale: e.g., “mothers should work only if necessary’), and the degree to which gender is perceived as a binary construct (5-item gender transcendence subscale: e.g., “people can be both aggressive and nurturing regardless of sex”). Higher scores on this measure are indicative of traditional gender role attitudes. The total scale was used here, demonstrating good internal consistency (α = .86) in this sample.

##### Attendance Intention

Football attendance intentions were evaluated using a scale adapted from Cunningham and Kwon (2003) that measured participants’ intentions to attend live football matches for both women’s and men’s AFL. This scale evaluates intentions and plans for attending sport events (e.g., “I intend to attend a [women’s/men’s] AFL game during the season”). Reliability analyses demonstrated excellent internal consistency (AFLW: α = .97; AFLM: α = .97) in this sample.

### Data analytic approach

Data were exported from the survey platform to IBM SPSS Statistics v28, where initial cleaning, coding, and assumption checks were completed. Descriptive analyses were conducted to investigate the nature of football fandom in this sample, and the main variables of interest. Pearson correlation analyses were run each condition (i.e., vignette (good vs. bad) and vignette (woman vs. man)) to explore relationships between the main variables of interest. Given our primary interest was in examining differences in participants’ perceptions based on player gender, the vignette conditions were collapsed into two larger groups (i.e., women players and men players). The analysis comprised two steps: exploratory analyses followed by hypothesis testing. Initially, a series of independent samples *t*-tests assessed differences in consumer intentions to watch AFLW and AFLM across genders. Additionally, a series of three-way ANOVAs were conducted to explore differences in skill ratings and performance ratings in relations to the AFLW/AFLM player, across genders and vignette conditions. Hypothesis testing was then conducted through multi-group path analysis to investigate the relationships among player evaluations, gender role attitudes, sport and masculinity attitudes, and sports consumer attitudes, as well as to test for invariance between the woman vignette and man vignette. Path analysis is a statistical approach used to evaluate the effects of observed variables on an outcome across multiple pathways (Hong et al., 2017; Valenzuela & Bachmann, 2017). The multi-group method was utilised to explore differences in the paths according to groups to identify invariance. Invariance testing involved freeing parameters, by assessing the configural and then metric invariance across the two conditions. The configural model assesses the pattern of associations between the observed variables and serves as the baseline, while metric invariance assesses the similarities in the paths.

The researchers first designed the conceptual model that informs the multigroup path analysis invariance testing based on prior research (Figure 1), with model fit guiding subsequent adjustments. The age and gender variables were treated as covariates within the model. Model fit was assessed using the following indices: CMIN/DF (< 3), CFI (> 0.95 indicating good fit), NFI (> 0.9), SRMR (< 0.05), and RMSEA (< 0.08) (Hooper et al., 2008; Hu & Bentler, 1999; MacCallum et al., 1996). The difference between models was assessed by change in CFI, and statistical significance for the individual paths were demonstrated by *p* values < .05.

## Results

### Descriptive statistics

Descriptive analysis was performed with the main variables of interest (see Table 2). Exploratory analyses were also conducted to contextualise levels of football fandom in the final sample. When the general fandom item was dichotomised, the majority of participants (88.2%) identified as fans of football as a sport. In reference to the specific leagues, 53.7% of participants reported following only the AFLM, while 34.2% stated they follow both the AFLW and AFLM equally. A smaller subset (1.5%) reported following only the AFLW, and 10.5% of participants did not follow either the AFLW or the AFLM. A small proportion of the sample (1.3%) still identified as football fans despite not following either league, suggesting broader engagement with the sport beyond the professional context.

**Table 2.** Descriptive statistics of main variables.

Variable	Condition 1 *		Condition 2		Condition 3		Condition 4		Total sample	
	Mean	*SD*	Mean	*SD*	Mean	*SD*	Mean	*SD*	Mean	*SD*
SRQ^a^	2.36	0.80	2.36	0.80	2.42	0.90	2.34	0.75	2.37	0.81
SMS^b^	2.01	0.85	2.02	0.84	2.03	0.98	1.98	0.82	2.01	0.88
Intention to attend:										
AFLW	2.87	1.64	2.95	1.65	3.27	1.81	3.06	1.80	3.04	1.74
AFLM	4.59	1.60	4.51	1.58	4.56	1.52	4.57	1.62	4.56	1.58
Desire to watch:										
AFLW	3.26	1.27	3.24	1.29	3.52	1.42	3.27	1.32	3.32	1.33
AFLM	4.83	1.03	4.77	1.08	5.01	0.91	4.88	1.02	4.87	1.02
Skill	3.28	0.97	3.27	0.87	5.63	0.60	5.67	0.59	4.47	1.40
Performance	3.02	0.88	2.92	0.86	5.72	0.54	5.74	0.54	4.36	1.55

*Note*. This table presents the means and standard deviations for the key variables of interest in each condition, and the total sample. The range for all variables above is 1–6.

^a^Gender role attitudes; ^b^ attitudes towards sport and masculinity.

*Condition 1 = woman, mediocre; Condition 2 = man, mediocre; Condition 3 = woman, good; Condition 4 = man, good.

### Exploratory analysis

#### Consumer differences

Independent samples *t*-tests were run to evaluate the differences between women’s and men’s desire to watch AFLW, and intention to attend AFLW. It was found that women (*M* = 3.60, *SD* = 1.34) reported a significantly higher desire to watch AFLW than men (*M* = 3.16, *SD* = 1.29), 95% CI [0.25, 0.62], *t*(824) = 4.571, *p* < .001, *d* = 0.33. Women (*M* = 3.47, *SD* = 1.81) also rated significantly higher on their intention to attend AFLW matches than men (*M* = 2.80, *SD* = 1.64), 95% CI [0.42, 0.91], *t*(813) = 5.353, *p* < .001, *d* = 0.40. In reference to men’s football, no differences were found between women’ (*M* = 4.62, *SD* = 1.64) and men’s (*M* = 4.51, *SD* = 1.55) intention to attend AFLM scores, 95% CI [−0.11, 0.34], *t*(813) = 0.696, *p* = .16, *d* = 0.06. Furthermore, no differences were found between women’s (*M* = 4.85, *SD* = 1.15), and men’s (*M* = 4.87, *SD* = 0.96) desire to watch AFLM, 95% CI [−0.16, 0.12], *t*(822) = −.293, *p* = .38, *d* = 0.01.

#### Performance and skill evaluation

As part of the exploratory analysis, reduced three-way between-groups ANOVAs were conducted to explore differences in performance and skill ratings of AFLM/AFLW players by main effects of participant gender, vignette (good vs. bad) and vignette (woman vs. man), and one interaction effect between vignette (good vs. bad) and vignette (woman vs. man). As can be seen in Table 3a and 3b, there were significant main effects for participant gender and vignette (good vs. bad) on both AFLM/AFLW player performance ratings and AFLM/AFLW player skill ratings (*p* < .05), however, no significant main effect of vignette (woman vs. man) was found for performance (*p* = .089) or skill (*p* = .633). Women rated players’ performances (*p* < .05), and players’ skills (*p* < .05) as higher compared to men. Additionally, performances (*p* < .001) and skills (*p* < .001) of AFLW/AFLM players were rated significantly higher in the good vignette compared to the bad vignette. Finally, the interaction effect between vignette (good vs. bad) and vignette (woman vs. man) was not significant for performance (*p* = .201) or skill (*p* = .720). As the interaction effects were not significant, no post hoc pairwise comparisons were conducted.

**Table 3a.** Three-way ANOVA results for performance ratings of AFLM/AFLW players by participant gender, vignette gender, and vignette context.

Variable	Levels	Mean	SD	ANOVA result
Gender^a^				*F*(1, 849) = 3.62, *p* = .037, *η*^*2*^*p* = .00 (very small)^c^
	Women	4.45	1.52	
	Men	4.42	1.56	
Vignette Gender^b^				*F*(1, 849) = 2.89, *p* = .089, *η*^*2*^*p* = .00 (very small)
	Woman	4.48	1.51	
	Man	4.37	1.58	
Vignette Context				*F*(1, 849) = 2532.85, *p<*.001, *η*^*2*^*p* = .73 (large)
	Good	5.71	0.60	
	Bad	3.03	0.94	
Vignette Gender X Vignette Context				*F*(1, 849) = 1.65, *p* = .201, *η*^*2*^*p* = .00 (very small)
	Women – Good	3.11	0.96	
	Men – Good	2.96	0.92	
	Women – Bad	5.72	0.55	
	Men – Bad	5.70	0.65	

*Note*. This table demonstrates the ANOVA outputs for performance ratings.

^a^Represents average scores in how women and men participants rated players across all conditions.

^b^Represents average ratings of total women players compared to ratings of total men players across the whole sample.

^c^*η*^*2*^*p* = Partial eta squared. Effect size interpretation guided by Field (2013).

**Table 3b.** Three-way ANOVA results for skill ratings of AFLM/AFLW players by participant gender, vignette gender, and vignette context.

Variable	Levels	Mean	SD	ANOVA result
Gender^a^				*F*(1, 849) = 11.65, *p* < .001, *η*^*2*^*p* = .01 (small)^c^
	Women	4.61	1.37	
	Men	4.47	1.42	
Vignette Gender^b^				*F*(1, 849) = 0.23, *p* = .633, *η*^*2*^*p* = .00 (very small)
	Women	4.55	1.40	
	Men	4.49	1.41	
Vignette Context				*F*(1, 849) = 1707.28, *p* < .001, *η*^*2*^*p* = .67 (large)
	Good	5.62	0.66	
	Bad	3.33	0.95	
Vignette Gender X Vignette Context				*F*(1, 849) = 0.13, *p* = .720, *η*^*2*^*p* = .00 (very small)
	Women – Good	3.36	1.02	
	Men – Good	3.30	0.90	
	Women – Bad	5.63	0.60	
	Men – Bad	5.62	0.73	

*Note*. This table demonstrates the ANOVA outputs for skill ratings.

^a^Represents average scores in how women and men participants rated players across all conditions.

^b^Represents average ratings of total women players compared to ratings of total men players across the whole sample.

^c^*η*^*2*^*p* = Partial eta squared. Effect size interpretation guided by Field (2013).

##### Analysis of AFLM Fan Sub-Sample

Further analysis was conducted for the subsample that reported watching AFLM exclusively, with reduced three-way between-groups ANOVAs to explore differences in performance and skill ratings of AFLM/AFLW players. This additional analysis was grounded in the assumption that participants that follow both leagues (or only the AFLW) are more likely to hold favourable attitudes towards women players. The reduced three-way ANOVAs explored the main effects of participant gender, vignette (good vs. bad) and vignette (woman vs. man), and one interaction effect between vignette (good vs. bad) and vignette (woman vs. man). Consistent with the prior results using the whole sample, significant main effects were found for participant gender and vignette (good vs. bad) for both performance (*p* < .05), and skill (*p* < .05) ratings, and no significant main effect for vignette (woman vs. man) (refer to Table 4a and 4b). Women followers of AFLM rated the performances and skills of players significantly higher than men (*p* < .05). performance ratings and skills ratings were significantly higher in the “good” vignettes compared to the “bad” vignettes (*p* < .001). Additionally, the interaction effect between vignette (good vs. bad) and vignette (woman vs. man) onto performance ratings was significant (see Table 4a). However, the interaction effect was not significant for skill ratings, and as such was not explored further with post hoc pairwise comparisons.

**Table 4a.** Three-way ANOVA results for performance ratings of players by participant gender, vignette gender, and vignette context in the AFLM fan subsample.

Variable	Levels	Mean	SD	ANOVA result
Gender^a^				*F*(1, 457) = 7.89, *p* = .005, *η*^*2*^*p* = .02 (small)^c^
	Women	4.40	1.48	
	Men	4.37	1.63	
Vignette Gender^b^				*F*(1, 457) = .61, *p* = .434, *η*^*2*^*p* = .00 (very small)
	Women	4.35	1.55	
	Men	4.42	1.62	
Vignette Context				*F*(1, 457) = 1660.11, *p <* .001, *η*^*2*^*p* = .78 (large)
	Good	5.76	0.50	
	Bad	2.96	0.94	
Vignette Gender X Vignette Context				*F*(1, 457) = 6.22, *p* = .013, *η*^*2*^*p* = .01 (small)
	Women – Good	3.02	1.01	
	Men – Good	2.91	0.86	
	Women – Bad	5.56	0.60	
	Men – Bad	5.87	0.40	

*Note*. This table demonstrates the ANOVA outputs for performance ratings with the subsample of participants who only follow men’s football.

^a^Represents average scores in how women and men participants rated players across all conditions.

^b^Represents average ratings of total women players compared to ratings of total men players across the whole sample.

^c^*η*^*2*^*p* = Partial eta squared. Effect size interpretation guided by Field (2013).

**Table 4b.** Three-way ANOVA results for skill ratings of players by participant gender, vignette gender, and vignette context in the AFLM fan subsample.

Variable	Levels	Mean	SD	ANOVA result
Gender^a^				*F*(1, 457) = 7.59, *p = *.006, *η*^*2*^*p* = .02 (small)^c^
	Women	4.49	1.38	
	Men	4.43	1.48	
Vignette Gender^b^				*F*(1, 457) = 2.99, *p* = .089, *η*^*2*^*p* = .01 (very small)
	Women	4.38	1.46	
	Men	4.51	1.44	
Vignette Context				*F*(1, 457) = 1167.07, *p* < .001, *η*^*2*^*p* = .72 (large)
	Good	5.65	0.50	
	Bad	3.20	0.94	
Vignette Gender X Vignette Context				*F*(1, 457) = 2.20, *p* = .139, *η*^*2*^*p* = .01 (very small)
	Women – Good	3.19	1.05	
	Men – Good	3.22	0.82	
	Women – Bad	5.54	0.65	
	Men – Bad	5.77	0.50	

*Note*. This table demonstrates the ANOVA outputs for skill ratings with the subsample of participants who only follow men’s football.

^a^Represents average scores in how women and men participants rated players across all conditions.

^b^Represents average ratings of total women players compared to ratings of total men players across the whole sample.

^c^*η*^*2*^*p* = Partial eta squared. Effect size interpretation guided by Field (2013).

Using pairwise comparisons with a Bonferroni correction for performance ratings, AFLM followers rated the performances of women in the bad vignettes as significantly poorer than the men in the bad vignette (*t*(457) = 2.33, *p* = .020, *d* = 0.22; small effect). However, no differences were found between the women players and the men players in the good vignettes (*t*(457) = 1.20, *p* = .231, *d* = 0.06; very small effect). Furthermore, in the men vignette, significant differences were reported between the good players and the bad players (*t*(457) = 30.14, *p* < .001, *d* = 2.82; large effect). This was also reflected in the women vignette (*t*(457) = 27.50, *p* < .001, *d* = 0.57; large effect). No other significant main effects or interaction effects were found.

##### Hypothesis Testing: Multigroup Path Analysis

As part of the hypothesis testing, a multigroup path analysis was run in AMOS v29 for the proposed model (see Figure 1) across two vignette gender groups: participants who evaluated a woman player (*n* = 395) and those who evaluated a man player (*n* = 406). Both groups reached acceptable model fit, indicating a close fit between the proposed model and the data (refer to Table 5), demonstrating it is appropriate to proceed with multigroup path analysis.

**Table 5.** Goodness-of-fit for multigroup invariance testing for the proposed model for the women vignette group and the men vignette group.

	CFI^a^	NFI^b^	SRMR^c^	RMSEA^d^	PCLOSE^e^	df	χ ^2^	Comparative Model	Δ(df)χ ^2^	ΔCFI
Women	.97	.96	.059	.07	.07	12	37.03			
Men	.95	.93	.059	.07	.08	12	36.71			
M1 Configural Model	.96	.95	.059	.05	.43	24	73.74			
M2 Path Model	.94	.92	.102	.06	.07	32	123.01	M2 v M1	(8) 49.27, *p* < .001	.02

*Note*. This table presents the fit indices and invariance testing for each model.

^a^Comparative Fit Index; ^b^ Normed Fit Index; ^c^ Standardised Root Mean Square Residual; ^d^ Root Mean Square Error of Approximation; ^e^ Probability of close fit.

#### Invariance testing

The first and least stringent model where the parameters were free to vary across the two groups, the configural model, had acceptable model fit as the fit indices met their cut-off criteria, with the exception of χ^2^ (see Table 5). However, χ^2^ is sensitive to sample size, in that the likelihood of reaching significance is greater in large sample sizes (Shi et al., 2019), leading to the erroneous conclusion of poor model fit. Therefore, additional fit indices are reported in Table 5. The second model, which constrained all regression paths to be equal across groups (i.e., path model), was approaching acceptable fit but this has significantly worsened from the configural model, indicating variance in the relationships across groups. As the path model was a significantly poorer fit, indicating group variances, no further models were tested.

The pathways that were variant across the woman vignette and man vignette groups were identified by systematically releasing the equality constraints to allow them to be free to vary. Significant differences were found in the path from sport masculinity attitudes to desire to watch football (∆ χ ^2^ /1df = 38.53, *p* < .001), and from sport masculinity attitudes to intention to attend AFL matches (∆ χ ^2^ /1df = 19.48, *p* < .001). The paths that were invariant across the woman vignette group and the man vignette group are discussed below.

#### Direct pathways

**Figure 3.**
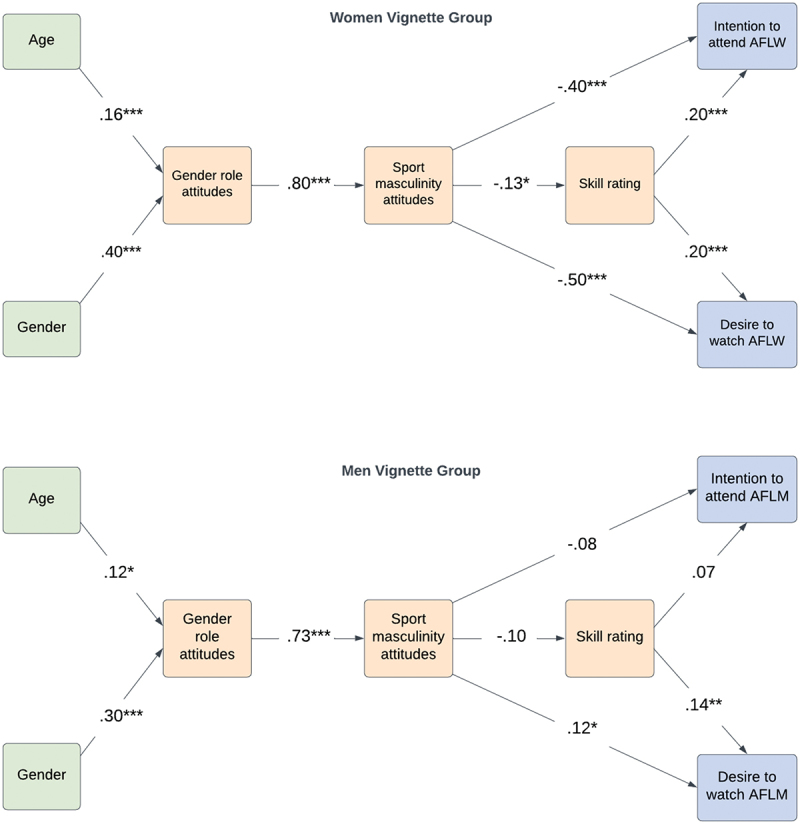
Final multi-group path models.

*Note*. This figure depicts the standardised direct path coefficients for the model in the women vignette group and the men vignette group. Green represents covariates, and orange represents exogenous/endogenous variables, and blue represents endogenous variables only.

The standardised path coefficients for each model are presented in Figure 3. For both groups, there was a significant positive relationship between gender role attitudes and sport masculinity attitudes supporting H1.

* *p* < .05, ** *p* < .01, *** *p* < .001.

In the women vignette group, there was a significant negative relationship between sport masculinity attitudes and skill evaluation, supporting H2. In the man vignette group, sport masculinity attitudes were not significantly linked to skill evaluation. Consistent with H3a, skill ratings were significantly positively associated with a desire to watch football in both models (i.e., AFLW and AFLM respectively). For both groups, there were significant negative associations between sport masculinity attitudes and desire to watch football, supporting H3b. Skill evaluation was also significantly positively linked to intention to attend AFLW matches (H4a) in the woman vignette group, but not for the man vignette group. Finally, there was a significant negative association between sport masculinity attitudes and intention to attend AFLW in the women vignette group (H4b), but this was not reflected in the men vignette group.

#### Indirect pathways

##### Woman Vignettes

The indirect pathways from gender role attitudes to both intention to attend and desire to watch a football match, via attitudes towards sport and masculinity, were significant in the woman vignette group (see Table 6). That is, with conservative gender role attitudes, individuals report more conservative attitudes towards sport and masculinity, and lower skills levels, and consequently lower intentions to attend and football match, and lower desire to watch a football match.

**Table 6.** Indirect pathways from the proposed model for the women vignette group using bootstrapping (*n* = 5000).

Pathway	β (SE)	Bias-corrected 90% CI [LL, UL]	*p*
SRQ ^a^ → SMS ^b^ → ITA	−.60 (.10)	[−.71, −.43]	.001
SRQ→ SMS→ DWS	−.60 (.10)	[−.70, −.44]	.001
SRQ→ SMS→ skill	−.10 (.04)	[−.17, −.04]	.009
SRQ→ SMS→ skill→ ITA	−.30 (.04)	[−.40, −.22]	.001
SRQ→ SMS→ skill→ DWS	−.40 (.05)	[−.44, −.30]	.001

^a^Gender role attitudes; ^b^ attitudes towards sport and masculinity.

##### Man Vignettes

Given the paths from attitudes towards sport and masculinity to perceived skill, and to intention to attend were non-significant in the man vignette group, indirect paths were not tested for these variables. The indirect pathway from gender role attitudes to desire to watch a football match via attitudes towards sport and masculinity was significant (see Table 7). The indirect pathway from gender role attitudes to desire to watch a football match was also significant via attitudes towards sport and masculinity and perceived skill level. That is, consistent with the woman vignette group, more conservative gender role attitudes correspond with more conservative attitudes towards sport and masculinity, and lower perceived skill levels, and consequently lower desire to watch.

**Table 7.** Indirect pathways from the proposed model for the men vignette group using bootstrapping (*n* = 5000).

Pathway	β (SE)	Bias-corrected 90% CI [LL, UL]	*p*
SRQ ^a^ → SMS ^b^ → DWS	−.12 (.05)	[−.20, −.03]	.024
SRQ→ SMS→ skill→ DWS	−.13 (.05)	[−.21, −.04]	.013

^a^Gender role attitudes; ^b^ attitudes towards sport and masculinity.

## Discussion

Building on prior research highlighting disparities in the consumption of women’s and men’s sport, the aim of this study was to investigate gender bias in consumer perceptions of athlete quality in football. The broader findings reflected the issue of consumption disparities in football in Australia, with general consumer attitudes across the sample being more positive towards AFLM compared to AFLW. This was further supported by the finding that women were more likely to want to watch and attend AFLW than men, which could be explained by previous research emphasising differences in consumer motivation between women’s and men’s sport more broadly (Guest & Luijten, 2018). For example, women may be more motivated to engage with women’s sport as a form of activism, identity, and empowerment (Allison et al., 2024; Giachino et al., 2024), which is unlikely to simultaneously motivate engagement from men. These patterns of consumption and engagement provide important context for understanding how gender bias may manifest in perceptions of athlete quality. However, it is also important to emphasise the limited timeframe for the development of AFLW fan attachment – particularly among groups less inclined to seek out women’s sport proactively. While current disparities in perceptions and engagement are important to document, they should also be interpreted in the context of a league that has not yet had the same historical opportunity to cultivate broad, multigenerational fanbases.

### Gender bias in perceptions of athlete quality

A preliminary exploration of the data revealed no significant differences in how participants rated the quality of the vignette players based on participant gender, or player gender. However, among participants who exclusively followed men’s football (i.e., did not follow AFLW), the skill and performance of men players was rated significantly higher than that of women players. This corresponds with findings from Gomez-Gonzalez et al. (2024), demonstrating men participants were more likely to rate the performance of women soccer players poorer than men soccer players. Similarly, Fink et al. (2016) found that positive perceptions of women’s athletic abilities remained constrained by gender essentialist stereotypes. These patterns support the notion that evaluations of women in sport may be heavily influenced by beliefs about women’s physical inferiority. Notably, this finding was only observed in the current study in vignettes featuring bad players, suggesting that men’s poor performance is not rated as harshly as women’s. This harsher evaluation of women’s performance could reflect the tendency for men’s poor performance to be attributed to external factors, whereas women’s failures are more likely seen as evidence of innate inferiority (Fink, 2015; Messner et al., 1993). Moreover, the performance of the mediocre woman athlete may have aligned more closely with stereotypes about the physical abilities of women, thereby prompting more negative evaluations. These findings emphasise the potential harm of stereotypes that frame women athletes as inadequate, which may ultimately hinder the continued growth of women’s sport.

### Gender norms, masculinity, and football consumption

The initial findings also suggested that biases in performance evaluations may be the product of a more complex interaction between gender attitudes and attitudes towards sport and masculinity. Accordingly, a pathway model was tested to examine the variables of interest in relation to consumer behaviour, with multigroup path analysis implemented to compare differences in participant attitudes for women’s and men’s football, respectively. The path model’s good fit to the data indicated that the relationships among these variables were well-represented within the model (West et al., 2012), supporting its utility in understanding the dynamics of consumer attitudes and behaviour in this context.

Examination of the findings revealed that conservative gender role attitudes were associated with men and older adults in both groups, aligning with previous findings (Glazbrook & Webb, 2024; Hickson & Marshan, 2023). The results also demonstrated that conservative sport and masculinity attitudes were significantly associated with lower ratings of skill for AFLW players, but not for AFLM players. Consistent with prior research in sport and gender (Gomez-Gonzalez et al., 2024; LaVoi & Dutove, 2012), this finding suggests that negative beliefs regarding the involvement of women in masculine sports can impact perceptions of women’s sporting ability. Such ideas were complemented by the multigroup analysis results, which demonstrated that conservative sport attitudes were significantly more strongly related to lower engagement and attendance of the AFLW, compared to the AFLM. These outcomes are demonstrative of the role of conservative gender attitudes (e.g., essentialist beliefs) in contributing to discrepancies in fan behaviour and support, hindering engagement with women’s sports leagues.

Furthermore, lower skill ratings were associated with decreased desire to watch both AFLW and AFLM, supporting the athlete quality as a broader motivation of sports consumers (Funk et al., 2022; Malchrowicz-Mośko & Chlebosz, 2019; Rizvandi et al., 2019). In contrast, intention to attend was only associated with skill ratings in relation to the AFLW, supporting the conception that beliefs about the athletic prowess of women have a stronger influence in shaping consumer behaviour in women’s sport compared to men’ sport (Allison & Pope, 2021). This finding suggests that athlete quality in isolation may be less critical for consumer motivation in men’s sport, as societal norms are sufficient to enforce the belief that men are the pinnacle of athleticism (Lebel & Danylchuk, 2009).

### Theoretical and practical implications

Beyond supporting prior research, our findings offer important theoretical insight into how gender bias may operate in the context of sports consumption, contributing to ongoing conceptual debates about the professionalisation of women’s sport and the persistence of essentialist logics that undervalue women’s athletic competence. In particular, these findings emphasise the potential influence of gender essentialist beliefs and stereotypes on perceptions of athlete quality and consumer behaviour. As Allison (2020) notes, essentialist ideologies offer a powerful framework to naturalise gender bias and differential treatment of women in sport. This is problematic as differences in investment between women’s and men’s sporting leagues may perpetuate a cycle of inequality justified by beliefs surrounding the ostensible physical inferiority of women – contributing to perceptions of women’s sport as less valuable (Gemar et al., 2025;). Such beliefs are facilitated by conservative attitudes towards gender, masculinity, and sport (Pope et al., 2022), demonstrating that the expansion of women’s football may be limited more by stereotypes and biases, rather than objective evaluations of athletic ability.

Where essentialist beliefs persist, there is also a failure to acknowledge the cultural and historical constraints that have limited the development of women’s sport more broadly, leaving women athletes operating at a systemic disadvantage. For example, Taylor et al. (2022) highlighted the tension faced by women athletes who must meet consumer and stakeholder expectations while navigating structural challenges such as financial strain and balancing dual careers. These insights emphasise the need to critically examine the logics underpinning the professionalisation of women’s sport, and to expand sport management research to address socio-cultural as well as economic and logistical drivers of inequality.

Our findings also challenge the suggestion that inadequate investment in women’s sport is justified by supposed lower quality and emphasise the need for revised approaches to the marketing and visibility of the AFLW. Whilst perceived athlete quality remains an important motivator for sports consumption, Allison and Pope (2021) caution that the further commercialisation of women’s sports risks eroding the unique motivational factors that attract its current audience (e.g., connection to players, inclusive fan environments). To navigate this tension, sports marketers should prioritise strategies that highlight the AFLW’s social and community-centred elements, whilst aiming to challenge the gender norms that lead to negative stereotyping about the athletic capacity of women. For example, the success of the Matildas in the 2023 World Cup was often met with responses such as “they’re actually good” (MacFarlane, 2023). Although, the phrasing insinuates the persistence of beliefs that women are not suited to elite sport, it also suggests that adequate exposure and media framing holds the potential to begin to challenge such stereotypes. As such, increased inclusion in primetime broadcasting and an extended AFLW season may ensure greater visibility and accessibility of women’s football, which may cultivate greater interest in the league (Marceau et al., 2025). Furthermore, engaging men as advocates for women’s sport, through cross-league support from AFLM players may further normalise interest in the AFLW and allow fans to view women players as equally valuable (Change Our Game & La Trobe University, 2023; Heffernan, 2024), whilst challenging the pervasive gender hierarchy in football and sport more broadly.

### Limitations and future research

The current study was not without limitations, which should be considered in the interpretation of the findings. First, whilst the use of vignettes provided a novel approach to the research topic, it may have resulted in inconsistent interpretations of athlete quality between participants. Additionally, factors such as inattention, unintentional priming, or insufficient information in the vignettes, may have influenced participants’ ratings, which could explain the lack of differences found between groups in the initial analysis. Moreover, the context of the research and reliance on convenience sampling potentially resulted in a sample with an inflated proportion of participants who held positive attitudes about gender and the AFLW, limiting insight into the attitudes of those with more conservative gender ideologies. Relatedly, education level was not included as a demographic variable and may have allowed for additional insight into the interplay between gendered attitudes and sports consumption. This study is also unable to fully address or contextualise public commentary that links perceived low performance in the AFLW to the league’s rapid expansion (see for example, Rucci, 2022; Taylor et al., 2022). While such claims are not always empirically substantiated, they may influence public perceptions of quality and merit further exploration.

Future researchers should consider implementing more robust of measures consumer perceptions of athlete quality in football to better understand the dynamics of gender bias in this context. This could be achieved through the use of videos (see for example, Gomez-Gonzalez et al., 2024), or live match settings to enhance the ecological validity of participant responses. Furthermore, qualitative research may provide richer insights into how consumers form and justify their attitudes towards women footballers and contextualise the role of essentialist ideology in shaping these views. Finally, given the league’s relative immaturity, longitudinal research could offer important valuable insight into how these attitudes change over time. This approach could be supported with secondary data analysis of AFLW-specific metrics (e.g., attendance figures, broadcast ratings) to generate a more comprehensive understanding of how perceptions of athlete quality translate into consumer behaviour. Such research would better capture the complexity of gender bias in sport and support the development of more targeted and effective strategies for change.

## Conclusion

Enhancing levels of fan interest and investment is essential to the further development and professionalisation of women’s sport leagues, such as the AFLW. Yet the abilities of women athletes continue to be underestimated, despite the growing participation of women in a diverse range of sports (Goorevich & LaVoi, 2024; Mohapatra, 2021). For women’s sports, these challenges are compounded by of a cycle of pervasive gender inequality characterised by barriers such as inconvenient game scheduling, and inadequate media coverage (Clargo & Skey, 2024; Cleland et al., 2020; Glazbrook et al., 2024). The findings of this study offer valuable insights into understanding the complexities of gender bias in the consumption of football in Australia, further supporting contemporary research which emphasises the need to challenge gender stereotypes in the marketing of sport. Furthermore, these outcomes call into question the validity of decisions by sport governing bodies and external organisations to provide inadequate funding and resources to women’s sport through the justification of perceived lower quality. To address these issues effectively, it is crucial to shift focus from solely blaming women athletes and consumers for lack of engagement, and instead hold key stakeholders accountable for enacting policy reforms that prioritise the expansion of women’s professional football and the development of an inclusive sporting industry.

## Supplementary Material

AppendixRevisions .docx

## Data Availability

Due to ethical limitations, the data associated with this research may be available from the corresponding author, upon reasonable request.

## Appendix

### Example Vignette: AFLM

On a cool Sunday afternoon, Sam stepped out to play his 50th game of professional football. Despite a light rain drizzle, the air was alive with energy as the excited home crowd waited in anticipation for the siren to sound, signalling the start of the first quarter. From the first bounce, Sam struggled to make any impact in defence of the ball. By the end of the first quarter, he had been outrun and left trailing behind as the ball was fed towards the oppositions’ goals multiple times. By half time, Sam scored his first goal for the game, but managed to turn the ball over to the opposition another two times. After marking the ball inside 50, Sam prepared for what should have been an easy set shot. Directly in front of goal, he took a few slow steps forward, before booting the ball towards the goals. From the moment the ball left his boot it was an obvious miss, with loud groans from the disappointed crowd echoing around the stadium as the ball veered to the left of the goals, for a behind. He went on to miss two more goals before three quarter time. There were some worrying signs in the fourth quarter when he landed awkwardly on his right leg with what appeared to be either a knee or calf injury, after going up for a contested mark. But after just a short time in the hands of the medical staff on field, he refused any further medical intervention, and continued playing. The nature of Sam’s injury was unconfirmed at the time of the game.
