# Supplementary material for: Not worth watching? Examining gender bias, perceptions of ability, and consumer behaviour for the Women’s Australian Football League
Source: Aust J Psychol. 2025 Aug 13;77(1):2543796. doi: 10.1080/00049530.2025.2543796 (PMC12351752; doi:10.1080/00049530.2025.2543796)
Supplement: AppendixRevisions .docx [file RAUP_A_2543796_SM9964.docx]

**Appendix**

**Example Vignette: AFLM**

On a cool Sunday afternoon, Sam stepped out to play his 50th game of professional football. Despite a light rain drizzle, the air was alive with energy as the excited home crowd waited in anticipation for the siren to sound, signalling the start of the first quarter. From the first bounce, Sam struggled to make any impact in defence of the ball. By the end of the first quarter, he had been outrun and left trailing behind as the ball was fed towards the oppositions’ goals multiple times. By half time, Sam scored his first goal for the game, but managed to turn the ball over to the opposition another two times. After marking the ball inside 50, Sam prepared for what should have been an easy set shot. Directly in front of goal, he took a few slow steps forward, before booting the ball toward the goals. From the moment the ball left his boot it was an obvious miss, with loud groans from the disappointed crowd echoing around the stadium as the ball veered to the left of the goals, for a behind. He went on to miss two more goals before three quarter time. There were some worrying signs in the fourth quarter when he landed awkwardly on his right leg with what appeared to be either a knee or calf injury, after going up for a contested mark. But after just a short time in the hands of the medical staff on field, he refused any further medical intervention, and continued playing. The nature of Sam's injury was unconfirmed at the time of the game.
